# Awareness, use and understanding of nutrition labels among children and youth from six countries: findings from the 2019 – 2020 International Food Policy Study

**DOI:** 10.1186/s12966-023-01455-9

**Published:** 2023-05-04

**Authors:** David Hammond, Rachel B. Acton, Vicki L. Rynard, Christine M. White, Lana Vanderlee, Jasmin Bhawra, Marcela Reyes, Alejandra Jáuregui, Jean Adams, Christina A. Roberto, Gary Sacks, James F. Thrasher

**Affiliations:** 1 School of Public Health Sciences, University of Waterloo, 200 University Avenue West, Waterloo, ON N2L 3G1 Canada; 2 École de Nutrition, Centre de Nutrition, Santé et Société (NUTRISS), Université Laval, 2425 Rue de L’Agriculture, Québec City, QC G1V 0A6 Canada; 3 School of Occupational and Public Health, Toronto Metropolitan University, 288 Church Street, Suite 300, ON M5B 1Z5 Toronto, Canada; 4 Instituto de Nutrición Y Tecnología de los Alimentos (INTA), Universidad de Chile, Avda. El Líbano, 5524 Macul, Santiago Chile; 5 Center for Health and Nutrition Research, Instituto Nacional de Salud Pública, Av. Universidad 655 Col, Santa María Ahuacatitlán, 62100 Cuernavaca, Mexico; 6 Centre for Diet and Activity Research (CEDAR), MRC Epidemiology Unit, Institute of Metabolic Science, University of Cambridge, Box 285, Cambridge, CB2 0QA UK; 7 Department of Medical Ethics and Health Policy, Perelman School of Medicine, University of Pennsylvania, 1121 Blockley Hall, 423 Guardian Drive, Philadelphia, PA 19104-6021 USA; 8 Global Obesity Centre, Institute for Health Transformation, Deakin University, 221 Burwood Highway, Burwood, Victoria 3125 Australia; 9 Department of Health Promotion, Education & Behavior, Arnold School of Public Health, University of South Carolina, 921 Assembly St, Columbia, SC 29208 USA; 10 Center for Population Health Research, Instituto Nacional de Salud Pública, Av. Universidad 655 Col, Santa María Ahuacatitlán, 62100 Cuernavaca, Mexico

**Keywords:** Nutrition labeling, Food policy, Comprehension, Adolescent

## Abstract

**Background:**

Nutrition facts tables (NFTs) on pre-packaged foods are widely used but poorly understood by consumers. Several countries have implemented front-of-package labels (FOPLs) that provide simpler, easier to use nutrition information. In October 2020, Mexico revised its FOPL regulations to replace industry-based Guideline Daily Amount (GDA) FOPLs with ‘Warning’ FOPLs, which display stop signs on foods high in nutrients of concern, such as sugar and sodium. This study examined self-reported awareness, use, and understanding of NFTs and FOPLs among young people in six countries with different FOPLs, with an additional focus on changes before and after implementation of Mexico’s FOPL warning policy.

**Methods:**

A ‘natural experiment’ was conducted using ‘pre-post’ national surveys in Mexico and five separate comparison countries: countries with no FOPL policy (Canada and the US), countries with voluntary FOPL policies (Traffic Lights in the UK and Health Star Ratings in Australia), and one country (Chile) with mandatory FOPL ‘warnings' (like Mexico). Population-based surveys were conducted with 10 to 17-year-olds in 2019 (*n* = 10,823) and in 2020 (*n* = 11,713). Logistic regressions examined within- and between-countries changes in self-reported awareness, use, and understanding of NFTs and FOPLs.

**Results:**

Across countries, half to three quarters of respondents reported seeing NFTs ‘often’ or ‘all the time’, approximately one quarter reported using NFTs when deciding what to eat or buy, and one third reported NFTs were ‘easy to understand’, with few changes between 2019 and 2020. In 2020, awareness, use and self-reported understanding of the Warning FOPLs in Mexico were higher than for NFTs in all countries, and compared with GDA FOPLs in Mexico (*p* < .001). Mandated Warning FOPLs in Mexico and Chile had substantially higher levels of awareness, use, and understanding than the voluntary Traffic Lights in the UK and Health Star Ratings in Australia (*p* < .001 for all).

**Conclusions:**

Mandated easy-to-understand FOPLs are associated with substantially greater levels of self-reported awareness, use and understanding at the population-level compared to NFT and GDA-based labeling systems.

**Supplementary Information:**

The online version contains supplementary material available at 10.1186/s12966-023-01455-9.

## Background

Nutritious diets are a critical component of childhood health [1, 2]. However, energy-dense and nutrient-poor diets are prevalent in many countries, in part due to increasing consumption of ultra-processed foods high in sugar, sodium, and saturated fat [3, 4]. As a result, the prevalence of overweight and obesity among children and adolescents has increased across a wide range of low-, middle-, and high-income countries [5, 6].

Nutrition labelling is a well-established policy measure for informing consumers about the contents of pre-packaged foods [7]. Virtually all countries have some form of nutrition facts tables (NFTs) on the ‘back’ or ‘side’ of packages, which usually display nutrient levels, energy content, and ingredient lists (see Supplemental Fig. 1). A wide range of studies demonstrate high levels of self-reported awareness and use of nutrition labelling among consumers, especially among females, younger and middle-aged adults, and consumers with healthier dietary intake [8, 9]. However, consumers have difficulty understanding and applying the quantitative information displayed in NFTs, with many unable to correctly calculate and apply nutrient amounts, serving sizes and percent daily values (a value intended to indicate how much a nutrient in a serving of food contributes to a daily diet) [8, 10]. Comprehension is particularly low among lower socioeconomic groups, which among other factors, may exacerbate socioeconomic disparities in dietary intake [11–14].

There is broad consensus that mandated nutrition labels should include more intuitive information that can be more easily understood by consumers with varying numeracy and literacy levels [15]. Accordingly, an increasing number of countries have implemented ‘front-of-package’ nutrition labels (FOPLs) [16–18]. FOPLs have two distinctive features: 1) they are located on the ‘front’ principal display area of food packages, and 2) they provide simple interpretive information that involve use of symbols and other summary indicators, as opposed to the numeric values presented in NFTs [19].

**Fig. 1:**
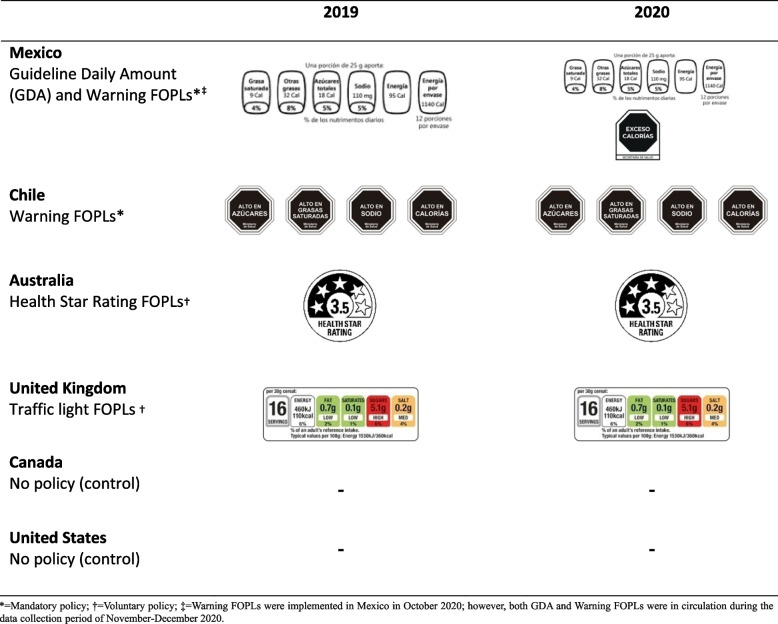
Front-of-package labels (FOPLs) evaluated in the International Food Policy Study, by country and year

Early versions of FOPLs were developed by the food industry, including the ‘Facts Up Front’ or Guideline Daily Amount (GDA) system. GDAs display information on key nutrients, such as sodium, saturated fat and sugars, specifying intake levels that typical healthy adults are guided to consume daily [20]. GDAs qualify as FOPLs to the extent that they appear on the front of packages; however, they convey the same quantitative nutrient information as NFTs rather than interpretative information common to other FOPL systems. In countries such as Canada and the United States (US), GDAs are commonly displayed on pre-packaged foods as part of voluntary industry-led initiatives; however, in 2014, Mexico endorsed GDA FOPLs as a national policy, mandating their display on pre-packaged foods [20]. More recently, governments have developed alternative FOPL systems, including the United Kingdom’s (UK) Traffic Light system, which uses green, yellow and red colouring to communicate ‘low’, ‘medium’ and ‘high’ nutrient levels [21]. The Health Star Rating in Australia and New Zealand provides ratings of the overall nutritional profile of packaged food from 0.5 to 5 stars [22], similar to the Nutriscore system, widely implemented in Europe, which rates foods on 5 levels using colours and a letter grade system [23]. Several countries have also implemented ‘Warning’-based FOPL systems, in which foods that exceed a certain threshold of energy, sugar, sodium, or saturated fat must display a stop sign or other cautionary symbols (see Fig. 1). Warning FOPLs were first implemented in Chile [24], followed by a number of other countries including Mexico, which replaced its mandatory GDA system in 2020 with the more interpretive Warning FOPLs [25].

An important factor in assessing the impact of FOPL policies is whether they are mandated to appear on all pre-packaged products (e.g., in Chile and Mexico) or whether FOPL policies are voluntary, in which case companies have the choice whether or not to display the government-endorsed systems on individual pre-packaged products. For example, the UK’s Traffic Light FOPLs and Australia’s Health Star Rating FOPLs are both endorsed by government, but voluntary in nature. A notable limitation of voluntary systems is that companies may selectively choose not to show FOPLs on products with less favourable nutritional profiles.

Research suggests that FOPLs are substantially easier for consumers to understand than traditional quantitively-based NFTs, particularly in identifying less healthy foods [26]. To date, there is no consensus on the most effective system; however, studies have consistently demonstrated that GDA-based FOPLs are less effective in helping consumers identify and select more nutritious foods compared to other FOPL systems (e.g., warning labels) [27]. Most evidence on FOPLs derives from experimental studies conducted within artificial study settings [27, 28] that fail to account for factors that influence the impact of nutrition labelling under ‘real-world’ conditions. Under actual conditions of use, consumers often experience time constraints and other attentional demands that make noticing and comprehension of labels more difficult [8, 9, 29]. The impact of nutrition labels can also be influenced by the implementation process for introducing new labels including accompanying public education campaigns, as well as wear-in and wear-out of FOPLs over time—in which the effectiveness of a FOPL increases with repeated exposure and greater consumer familiarity—and industry actions, such as the level of uptake for voluntary labelling policies [30]. To date, there is relatively little evidence on population-level outcomes following actual implementation of FOPLs, with the exception of several studies conducted to evaluate the Warning FOPLs implemented in Chile in 2016. One longitudinal study in Chile found improvements in the healthfulness of food purchases following implementation of Warning FOPLs, including substantial reductions in sugar-sweetened beverages; however, Chile implemented a range of other policy measures at the same time as the FOPLs (e.g., restrictions on marketing and school sales of products high in sugar, sodium, saturated fat and/or calories), such that the changes cannot be solely attributed to the FOPLs [31].

The vast majority of research on FOPLs has been conducted with adults. However, children and youth are an important target audience of nutrition labels given that food packaging can influence child preferences, and healthy dietary patterns among youth predict health dietary practices among adults [32–34]. Accordingly, many countries encourage parents to educate children on the use of nutrition labels when selecting foods [35]. Many young people also report using NFTs; however, like adults, youth struggle to understand and apply nutrient values [36–38]. Several experimental studies indicate that FOPLs can help young people to identify more nutritious foods, with the highest efficacy for Chilean-style Warning FOPLs [39–41]. Qualitative research and cross-sectional surveys conducted in Chile with children [42] and their parents [43] following implementation of the Warning FOPLs also suggests that children are broadly aware of the warnings. Self-reported use and noticing of the Chilean Warning FOPLs among parents of children was also associated with greater self-reported changes in buying labelled/unhealthy foods [44].

The current study sought to examine the ‘real world’ impact of national FOPL policies among young people using a ‘natural experiment’ research design. Natural experiments consist of comparisons between groups in the absence of randomization. In the case of national-level nutrition policies, countries serve as comparison groups and differences in dietary trends between countries with and without nutrition policies are the analytical focus [45, 46]. The current study examined differences in self-reported noticing (i.e., awareness), use, and understanding of nutrition labels among youth in six countries: Australia, Canada, Chile, Mexico, the UK, and the US. As part of the broader objective to understand differences in national-level FOPL policies, the study examined changes to Mexico’s FOPL policy, which occurred during the period of study. In October 2020, Mexico implemented a new FOPL policy and replaced the mandated GDA system that had been implemented in 2014 with Chilean style ‘High In’ Warning FOPLs [25]. Population-based surveys were conducted in Mexico before and after implementation of the FOPL warning policy, and in five ‘comparison’ countries in which no changes in FOPL policy were implemented over the same time period, producing within- and between-country comparisons over time. Comparison countries included two with no government-endorsed FOPL policy (Canada and the US), two with government-endorsed voluntary FOPL policies (Traffic Light FOPLs in the UK and Health Star Rating FOPLs in Australia), and one with a mandatory FOPL policy (‘High In’ warnings in Chile)—see Fig. 1. In addition to examining FOPL outcomes, self-reported noticing, use and understanding of NFTs were also assessed in each country to directly compare use of FOPLs and NFTs over time.

## Methods

Data were collected as part of the International Food Policy Study (IFPS) Youth Surveys, consisting of repeat cross-sectional surveys of youth aged 10–17 from six countries: Australia, Canada, Chile, Mexico, the UK, and the US. These countries were selected as they have broad similarities in food environments and cultures, but differ in national-level policies, including FOPL policies – both in terms of the existence of any FOPL policy and the type implemented. Youth were recruited to complete an online survey through parents/guardians enrolled in the Nielsen Consumer Insights Global Panel and their partners’ panels. Email invitations were sent to a random sample of adult panelists with a potentially eligible child. Only one child per household was invited. Parental consent was obtained; after screening for eligibility, children provided assent. The target sample size in Canada (*n* = 4,000) was higher than other countries to provide greater power for sub-national tests between provinces unrelated to the current analysis. The American Association for Public Opinion Research cooperation rate #2 was 76.8% in 2019 and 79.6% in 2020, calculated as the percentage of participants who completed the survey out of eligible participants who accessed the survey link [47].

Data collection occurred in November and December 2019 and 2020. Surveys were conducted in English in Canada, the US, the UK and Australia; Spanish in Mexico, Chile, and the US; and in French in Canada, with adaptations for country-specific terminology. The child’s parent/guardian received compensation according to their panel’s usual incentive structure (e.g., points-based rewards). The study was reviewed by and received ethics clearance through a University of Waterloo Research Ethics Committee (ORE# 41477). A full description of study methodology is available in the IFPS Technical Reports [48, 49].

### Measures

Consumer awareness and comprehension are fundamentally important factors for assessing the impact of nutrition labels and product labelling more generally [29, 50]. The extent to which individuals notice and cognitively process warnings is the most important determinant of memory and attitude change in response to new information, including in the context of health warnings [29, 50, 51]. Research on product labelling and health warnings in other consumer product domains has demonstrated that self-reported measures of noticing and use are associated with changes in knowledge and behaviour in experimental studies, and in prospective cohort studies conducted following implementation of national labelling polices [27, 29, 36, 50, 52]. Label noticing and use also represent ‘proximal’ measures of labelling policies in population-based studies: compared to outcomes such as dietary intake, which have a wide range of determinants, label noticing and use are more specific to the policies that are being evaluated, and are thus less likely to be affected by other confounding factors [53]. Overall, measures of noticing and use are key mediators for assessing the impact of labelling policies at the population level. 

#### Self-reported noticing and use of nutrition labels

Separate questions assessed noticing and use of NFTs (all countries), and noticing and use of FOPLs in the four countries in which FOPLs had been implemented (Australia, UK, Mexico and Chile). Respondents viewed an image of either a NFT or a FOPL from their respective country when responding to questions (see Fig. 1 and Additional File 1). *Noticing* was assessed by asking: “Have you seen this type of food label on packages or in stores?” with the response options on a 5-point scale ranging from, “Never / Rarely / Sometimes / Often / All the time”. *Use* was assessed by asking, “Do you use this type of food label when deciding what to eat or buy?”, with the same response categories. These measures were adapted from previous population-based studies [54]. Analytical models used binary versions of the *Noticing* and *Use* variables (0 = never/rarely/sometimes, 1 = often/all the time); sensitivity analyses were also conducted using the original 5-item scale treated as a continuous variable (see Additional Files 2, 3 and 4).

#### Self-reported understanding of nutrition labels

The primary rationale and objective of FOPL policies is to enhance consumer understanding of nutrition information for pre-packaged foods. While viewing the relevant NFTs, participants were asked: “Do you find this information… very hard to understand / hard to understand / in the middle / easy to understand / very easy to understand.” Participants in Australia, Chile, Mexico and the UK were also shown an image of a FOPL from their respective countries (Fig. 1) and asked to respond to the same measure of self-reported understanding. The 2020 data collection occurred in parallel with the transition from the GDA FOPL to the Warning FOPL in Mexico, which had an initial implementation date of October 2020 and a subsequent extension until December 2020. Food and beverage companies were allowed to use provisional stickers between October 2020 and March 2021 in order to help companies gradually comply with the new regulation, such that both FOPLs were still in circulation. Thus, all FOPL measures were asked separately in Mexico for the GDA and Warning FOPL, using the same wording but separate images of the relevant FOPL. Binary versions of the outcome variable were analyzed for *Label Understanding* (0 = very hard/hard/in the middle, 1 = easy/very easy). Sensitivity analyses were also conducted using the original 5-level response options and the findings were highly consistent; therefore, results are only shown for the dichotomous outcome.

#### Socio-demographic characteristics

Sociodemographic measures included age, sex-at-birth (male, female), and ethnicity. Ethnicity was assessed using country-specific race/ethnicity categories and analysed as a derived variable (majority/minority) to accommodate different measures across countries. Perceived income adequacy was assessed with the question “Does your family have enough money to pay for things your family needs?” (Not enough money/Barely enough money/Enough money/More than enough money) [55].

### Analysis

A total of 23,139 youth completed the IFPS surveys across the six countries in 2019–2020, of which 603 (2.6%) were removed due to missing data on the outcome variables (*n* = 40), or further missing data on income adequacy (*n* = 225), ethnicity (*n* = 309), or both (*n* = 29). The final sample included 22,536 respondents (2019 = 10,823; 2020 = 11,713). Post-stratification sample weights were constructed using a raking algorithm with population estimates in each country separately based on age group, sex, region, and (except in Canada) ethnicity. Weights were subsequently rescaled to each sample size.

Descriptive findings are reported for all outcomes, stratified by country. Separate logistic regression models were run for each primary outcome: NFT use, NFT awareness and NFT understanding with data from all six countries; and FOPL use, FOPL awareness, and FOPL understanding with data from the countries in which FOPLs had been implemented (Australia, UK, Mexico and Chile). For all outcomes, models were run in two steps: in step 1 the model included only the ‘main effect’ variables; in step 2, a two-way interaction between year x country was added to test differences between countries over time. All pairwise contrasts between countries are reported. Models were adjusted for age, sex at birth, ethnicity, and perceived income adequacy, and included indicator variables for country and survey year.

In the four countries that had implemented FOPL policies, repeated measures models were also conducted to directly compare NFT and FOPL awareness, use, and understanding. Models were stratified by country, only included 2020 data, and adjusted for the same sociodemographic correlates described above. A generalized estimating equations (GEE) model with an unstructured correlation matrix was used to account for the correlation between outcomes for different labels from the same individual using the GENMOD procedure in SAS version 9.4. All analyses are weighted unless otherwise noted and 95% confidence intervals are reported for adjusted odds ratios. Analyses were conducted using SAS v9.4 (SAS Institute Inc., North Carolina).

## Results

### Sample characteristics

Table 1 shows sample characteristic overall and by country.

**Table 1:** Sociodemographic characteristics among the overall sample and across countries, 2019–2020 (data are weighted % and means) (unweighted n) estimates, *n* = 22,536)

	**Overall** (*n* = 22,536) % (n)	**Canada** (*n* = 7,345) % (n)	**Australia** (*n* = 2,979) % (n)	**UK** (*n* = 2,975) % (n)	**US** (*n* = 3,147) % (n)	**Mexico** (*n* = 3,328) % (n)	**Chile** (*n* = 2,762) % (n)
**Age (years)**							
Mean (SD)	13.5 (2.2)	13.5 (2.3)	13.4 (2.2)	13.5 (2.1)	13.5 (2.2)	13.5 (2.2)	13.5 (2.2)
**Sex**							
Male	51.0 (11,825)	50.9 (3,751)	51.3 (1,602)	51.2 (1,468)	51.1 (1,611)	50.7 (1,861)	51.1 (1,532)
Female	49.0 (10,711)	49.1 (3,594)	48.7 (1,377)	48.8 (1,507)	48.9 (1,536)	49.3 (1,467)	48.9 (1,230)
**Ethnicity**							
Majority	73.9 (18,037)	72.4 (5,421)	75.4 (2,387)	82.8 (2,614)	51.9 (2,267)	79.1 (2,892)	85.2 (2,456)
Minority	26.1 (4,449)	27.6 (1.924)	24.6 (592)	17.2 (361)	48.1 (880)	20.9 (436)	14.8 (306)
**Perceived Income Adequacy**							
Not enough money	4.0 (8757)	2.8 (202)	4.4 (137)	4.4 (132)	4.6 (130)	4.4 (131)	5.5 (143)
Barely enough money	20.6 (4,514)	15.1 (1,097)	17.8 (540)	20.7 (623)	21.9 (632)	28.9 (905)	26.5 (717)
Enough money	62.2 (14,141)	63.1 (4,668)	63.6 (1,889)	63.2 (1,889)	57.0 (1,838)	61.7 (2,091)	63.3 (1,766)
More than enough money	13.2 (3,006)	19.0 (1,378)	14.2 (413)	11.8 (331)	16.5 (547)	5.0 (201)	4.7 (136)

*UK* United Kingdom, *US* United States

Ethnicity categories as per census questions asked in each country: 1) Canada majority = White, minority = other ethnicity; 2) Australia majority = only speaks English at home, minority = speaks a language besides English at home; 3) UK majority = White, minority = other ethnicity; 4) US majority = White, minority = other ethnicity; 5) Mexico majority = Non-indigenous, minority = indigenous; 6) Chile majority = Non-indigenous, minority = indigenous

### Noticing nutrition labels

#### NFT noticing

**Fig. 2:**
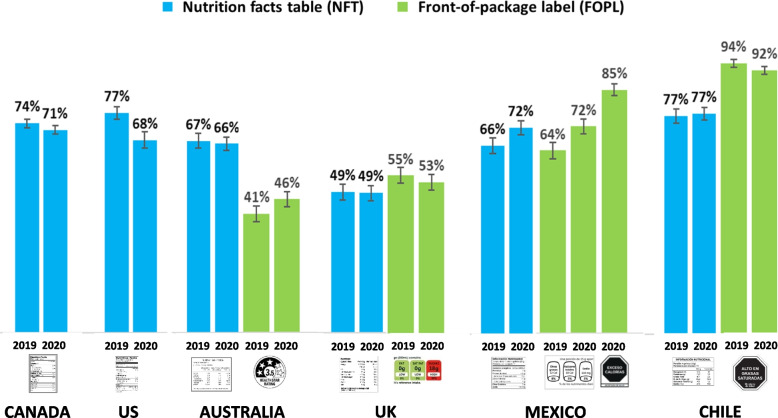
Percentage of respondents aged 10–17 who reported noticing food labels on packages or in stores ‘often’ and ‘all the time’ – by country and year (*N* = 22,536)

Figure 2 shows the percentage of respondents who reported seeing NFTs and FOPLs ‘often’ or ‘all the time’ on packages or in stores. Across both years, noticing NFTs was lowest in the UK compared to all other countries (all contrasts *p* < 0.001), and lower in Australia compared to the US, Canada, Mexico, and Chile (all contrasts *p* < 0.01). Between 2019 and 2020, noticing NFTs decreased in the US (AOR = 0.61, 0.51–0.74, *p *< 0.001) and Canada (AOR = 0.90, 0.81–1.00, *p* = 0.041), and increased in Mexico (AOR = 1.36, 1.14–1.63, *p* < 0.001), with no changes in the UK, Australia or Chile.

#### FOPL noticing

Across 2019 and 2020, noticing FOPLs was highest for the Warning FOPL in Chile compared to all other countries (*p* < 0.001 for all contrasts). In addition, noticing the GDA FOPL in Mexico was higher than the Health Star Rating FOPL in Australia (AOR = 0.34, 0.30–0.39, *p* < 0.001) and the Traffic Light FOPL in the UK (AOR = 0.53, 0.47–0.60; *p* < 001). Between 2019 and 2020, noticing increased for the GDA FOPL in Mexico (AOR = 1.52, 1.27–1.81; *p *< 0.001), and the Health Star Rating FOPL in Australia (AOR = 1.20, 1.03–1.40; *p* = 0.017), whereas noticing the Warning FOPL in Chile decreased (AOR = 0.67, 0.49–0.92; *p* < 0.014), with no change in the UK.

In 2020, Mexican youth were more likely to notice the new Warning FOPL than the GDA FOPL in 2019 (AOR = 3.40, 2.77–4.18; *p* < 0.001). The difference in noticing between the GDA FOPL in 2019 and the Warning FOPL in 2020 among Mexican youth was significantly greater than any changes in noticing FOPLs in any other country (all contrasts *p* < 0.001). 

#### Comparisons between NFT & FOPL noticing

In 2020, Mexican youth were more likely to notice the new Warning FOPL compared to the NFT (AOR=2.21, 1.84-2.66; p<.001) and the GDA FOPL (AOR=2.25, 1.87-271; p<.001), with no difference between the NFT and GDA FOPL. Youth were also more likely to notice FOPLs versus NFTs in the UK (AOR=1.16, 1.05-1.29, p=.004) and Chile (AOR=3.37, 2.75-4.12; p<.001). In contrast, Australian youth were less likely to notice the Health Star FOPL than NFTs (AOR=0.43, 0.38-0.49, *p*<.001). 

### Use of nutrition labels

#### NFT use

**Fig. 3:**
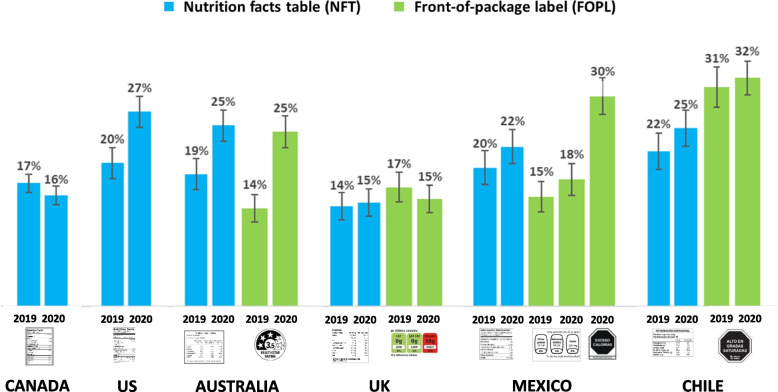
Percentage of respondents aged 10–17 who reported using nutrition labels ‘often’ or ‘all the time’ when deciding what to eat or buy – by country and year (*N* = 22,536)

Figure 3 shows the percentage of respondents who reported using NFTs and FOPLs ‘often’ or ‘all the time’ in deciding what to eat or buy in 2019 and 2020. Across both years, NFT use was lowest in the UK and Canada compared to all other countries (*p* < 0.001 for all contrasts). NFT use was higher in Chile than Australia (AOR = 1.19, 1.04 -1.35, *p* = 0.01) and Mexico (AOR = 1.17, 1.02–1.34, *p* = 0.028). Between 2019 and 2020, NFT use increased in Mexico (AOR = 1.24, 1.01–1.52; *p* = 0.041), the US (AOR = 1.47, 1.21–1.78; *p* < 0.001), and Australia (AOR = 1.46, 1.21–1.75; *p* < 0.001).

#### FOPL self-reported use

Across both years, FOPL self-reported use was higher for the Warning FOPL in Chile compared to all other countries (*p* < 0.001 for all contrasts), and higher in Australia versus the UK (AOR = 1.20, 1.04–1.39; *p* = 0.013). No changes in FOPL use were observed in any country, other than Australia, where use of Health Star FOPL increased between 2019 and 2020 (AOR = 1.98, 1.62–2.43; *p* < 0.001).

In 2020, use of the new Warning FOPL in Mexico was substantially higher than the GDA FOPL in 2019 (AOR = 2.43, 1.97–2.98; *p* < 0.001). In Mexico, the difference in use between the 2019 GDA FOPL and the 2020 Warning FOPL was significantly greater than changes in using FOPLs in Chile and the UK (*p* < 0.001 for all contrasts), but not Australia.

#### Comparisons between NFT & FOPL use

In 2020, Mexican youth were more likely to report using the new Warning FOPL compared to the NFT (AOR = 1.47, 1.28–1.68; *p* < 0.001) and the GDA FOPL (AOR = 1.98, 1.69–2.31; *p* < 0.001). In Chile, youth were also more likely to report using the Warning FOPLs than NFTs (AOR = 1.42, 1.26–1.60, *p* < 0.001), with no differences between NFT and FOPL use in the UK or Australia.

### Understanding nutrition labels

#### NFT understanding

**Fig. 4:**
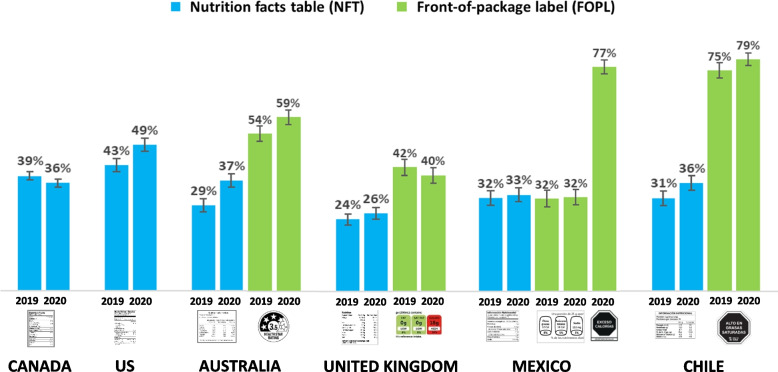
Percentage of respondents aged 10–17 who reported that nutrition label information was ‘easy’ or ‘very easy’ to understand – by country and year (*N* = 22,536)

Figure 4 shows the percentage of respondents who reported that NFTs and FOPLs are ‘easy’ or ‘very easy’ to understand. Across both years, self-reported NFT understanding was lowest in the UK and highest in the US compared to all other countries (*p* < 0.001 for all contrasts). Understanding NFTs was also higher in Canada versus Mexico (AOR = 1.12, 1.01–1.24; *p* = 0.035) and Australia (AOR = 1.15, 1.05–1.27, *p* = 0.003); and higher in Chile versus Australia (AOR = 1.13, 1.00–1.27; *p* = 0.043). Between 2019 and 2020, NFT understanding increased in Chile (AOR = 1.26, 1.06–1.50; *p* = 0.008), the US (AOR = 1.29, 1.09–1.53; *p* = 0.003), and Australia (AOR = 1.44, 1.22–1.70; *p* < 0.001).

#### FOPL understanding

Across both years, self-reported FOPL understanding was highest for the Warning FOPL in Chile compared to all other countries, and lowest for the GDA FOPL in Mexico (*p* < 0.001 for all contrasts). FOPL understanding was also higher for the Health Star FOPL in Australia than the Traffic Light FOPL UK (AOR = 1.86, 1.67–2.08; *p* < 0.001).

Between 2019 and 2020, FOPL understanding increased in Chile (AOR = 1.25, 1.04–1.51; *p* = 0.020) and Australia (AOR = 1.22, 1.04–1.42; *p* = 0.012), with no changes in understanding the GDA FOPL in Mexico or the UK.

In 2020, self-reported understanding of the new Warning FOPL in Mexico was substantially higher than the GDA FOPL in 2019 (AOR = 7.58, 6.29–9.14; *p* < 0.001). The increase in understanding the new Warning FOPL in Mexico was significantly greater than changes in understanding FOPLs in all other countries (*p* < 0.001 for all).

#### Comparisons between NFT & FOPL understanding

In 2020, Mexican youth reported greater understanding of the new Warning FOPL versus the NFT (AOR = 7.20. 6.15–8.43; *p* < 0.001) and the GDA FOPL (AOR = 7.40, 6.29–8.70; < 0.001), with no difference between the NFT and GDA FOPL. In Australia, Chile, and the UK, FOPLs were also rated as easier to understand than NFTs (*p* < 0.001 for all contrasts).

## Discussion

The current study represents the most comprehensive ‘real world’ study of nutrition labels among children and youth to date. The study has three primary findings. First, children and youth report widespread awareness and moderate levels of use and understanding of nutrition labels. Second, levels of awareness, use and understanding were strongly associated with the type of nutrition label. Most notably, the implementation of Warning FOPLs in Mexico led to considerable increases in awareness, use and understanding of nutrition labels. Third, mandated Warning FOPLs had greater impact than other FOPL systems, including the voluntary FOPL policies implemented in Australia and the UK. These findings are described in more detail below.

Although nutrition labels have traditionally been targeted at adult consumers, young people in all countries reported high levels of noticing labels. Across countries, half to three quarters of respondents reported seeing NFTs ‘often’ or’all the time’, whereas one quarter reported using NFTs when deciding what to eat or buy. The ratio of consumer use to awareness is similar to previous studies, in which approximately one quarter to one third of those who notice labels also report using this information to guide their food choices [56]. Few studies with children or youth are available for comparison, with the exception of a US study conducted in 2005/06, in which 17% of youth reported ‘reading’ NFTs [57]. In a parallel IFPS survey for adults, levels of use and understanding for both NFTs and FOPLs among youth respondents were approximately half that of adults from the same countries [58]. This is consistent with the general finding that use of labels increases with age and peaks in middle-age [8]. In our study, noticing and use of NFTs were generally stable within countries between 2019 and 2020, with the exception of increased use in the US and Australia in 2020. Increased use among US youth may reflect mandated changes to the NFTs implemented in 2020 (between IFPS data collection periods), including bolded and larger type faces for calories and serving size, as well as the inclusion of ‘added sugars’ in the nutrient list [59]. In addition, awareness and use of NFTs was notably lower among UK youth. The reasons for this are not entirely clear, although it is possible UK consumers are less likely to seek out the nutrient information in NFTs because nutrient values are also displayed in the Traffic Light FOPLs.

Self-reported understanding of NFTs was substantially lower than FOPLs in all four countries in which FOPLs were implemented, with one exception: Mexican youth were no more likely to report understanding the quantitatively-based GDA FOPLs than the NFTs, as discussed below. These findings are consistent with previous population-based data from Mexico [60], as well as previous research indicating low understanding of the quantitative information in NFTs among young people [38], and randomized controlled trials that use objective tests of comprehension, in which the use of colours or simple descriptors enhances understanding of numeric nutrient information [37].

Implementation of the Mexican Warning FOPLs in 2020 was associated with substantially higher self-reported levels of awareness, use, and understanding compared to NFTs in any country, as well as the GDA FOPLs that were mandatory in Mexico starting in 2014. In both 2019 and 2020, the GDA FOPLs in Mexico were the only FOPLs that were consistently the same or lower than NFTs in terms of awareness, use, and understanding. This is not surprising given that GDA FOPLs present similar quantitative nutrient information as NFTs. Compared to the GDA FOPLs, Mexican youth were approximately twice as likely to report using the Warning FOPLs and more than twice as likely to report that the Warning FOPLs were easy to understand. The current findings are consistent with a range of other studies indicating that the GDAs are the least effective FOPL system, [41, 61, 62] including experimental studies previously conducted in Mexico, which have found lower understanding of GDA versus Warning FOPLs [63–65], as well as weaker influence on hypothetical purchase tasks among low- and middle-income adults [66].

Levels of awareness, use, and understanding of the Warning FOPLs in Mexico were strikingly similar to the levels observed for the Warning FOPLs implemented in Chile. Both Warning FOPL systems outperformed the Health Star Ratings FOPLs in Australia and the Traffic Light FOPLs in the UK on all measures. For example, in 2020, 92% of respondents in Chile and 85% in Mexico reported seeing the Warning FOPLs ‘often or all the time’, compared to 46% in Australia and 53% in the UK. Differences in FOPL awareness likely reflects differences in mandated versus voluntary policies, which have suffered from low industry uptake. Despite efforts to encourage Australian manufacturers to display Health Star Ratings, the FOPLs are displayed on less than 50% of pre-packaged foods and remain less common among products with lower nutritional quality [30, 67, 68].

Warning FOPLs in Mexico and Chile were also substantially higher on the key outcome of self-reported understanding, which is the primary rationale for implementing FOPLs: almost 80% of youth in both Chile and Mexico perceived the Warning FOPLs as ‘easy’ or ‘very easy’ to understand, compared to 40%—59% in the UK and Australia, and 32% for the GDA FOPL previously implemented in Mexico. Although the current study used a self-reported measure of understanding, the findings are consistent with qualitative research [69] and experimental studies using objective measures of comprehension and functional tasks [41, 70]. Among the four countries that had implemented FOPLs, self-reported understanding was related to the amount of quantitative nutrient information presented in each FOPL system: self-reported understanding was lowest for FOPLs with the greatest focus on nutrient values—the GDA FOPLs in Mexico, which only provide nutrient-specific numbers, followed by the Traffic Light FOPLs in the UK, which include interpretive colours but retain a primary focus on nutrient amounts. Self-reported understanding was higher for the Health Star Rating FOPLs, which provide a single summary rating score, and highest for the Warning FOPLs in Mexico and Chile, which provide no quantitative information. While all FOPL systems other than the GDA outperformed NFTs on measures of comprehension, the findings highlight the importance of using simple, recognizable symbols such as the stop signs featured in Mexico and Chile’s Warning FOPLs, which are easily understood by children and consumers with lower literacy and numeracy levels [29, 71]. The visual design of Warning FOPLs is also consistent with industry marketing practices for child-oriented foods, which use symbols rather than quantitative nutrient information [72]. Future studies might examine whether FOPLs help to counteract child-directed marketing strategies on packaging of less healthy foods [73]. Warning FOPLs also differ from other systems in that they are inherently dissuasive in nature and may be most effective in helping consumers to identify and avoid less healthy foods, whereas other systems convey a broader nutrient profile [74]. The dissuasive nature of Warning FOPLs may also be most effective in encouraging manufacturers to reformulate products that exceed nutrient thresholds and trigger mandated Warning FOPLs [75]. Multinational food companies have strongly opposed Warning FOPLs in Chile, Mexico and a growing number of other jurisdictions [74, 76, 77]. In particular, the industry has lobbied against the use of ‘stop signs’ and other Warning-based symbols in favour of less effective GDA-based systems and more neutral symbols, such as the magnifying glass FOPL recently implemented in Brazil [70, 78].

### Limitations

This study is subject to limitations common to survey research. Respondents were recruited using non-probability based sampling; therefore, the findings do not provide nationally representative estimates. However, quota sampling and post-stratification weights were constructed using age group, sex, and region in all countries, as well as ethnicity in all countries except Canada. The prevalence of self-reported overweight and obesity was also similar between the IFPS samples and national benchmark surveys in each country [48, 49].

The primary outcomes were based on self-reported measures of label awareness, use, and understanding. As described above, noticing and use are well-established measures for assessing consumer labelling and have been shown to predict consumer behaviour in prospective cohort and experimental studies, including dietary intake among youth [27, 29, 36, 50, 52]. Self-reported understanding of nutrition labels is also associated with ‘objective’ functional tests of NFT understanding, with similar patterns when comparing subgroups or population-level differences across countries [79].

Other than the voluntary versus mandatory nature of national level FOPL polies, the current study provides limited analysis of other implementation factors that could moderate or enhance the impact of labelling policies. Most notably, implementation of the Mexican Warning FOPL was still in progress during data collection. At the time of data collection, no communication campaign had yet been implemented to promote awareness or use of the Warning FOPLs; similarly, other aspects of the policy, such as bans on the use of cartoon characters on the front of packages had not yet been implemented; thus, the current study may have underestimated changes after full implementation. Finally, it is unknown whether the COVID-19 pandemic may have affected consumer interactions with pre-packaged nutrition labels; however, the between-country study design helps to adjust for any common pandemic effects across countries.

## Conclusions

Comprehensive nutrition labelling on food packaging is one of several policy-level interventions with the potential to enhance dietary intake and prevent nutrition-related chronic disease. The findings from this large international study provide important ‘real world’ evidence on FOPL policies at the population-level using an innovative natural experiment design. The results are highly consistent with the previous evidence from randomized controlled trials and qualitative research: simple, easy-to-understand FOPLs serve as an effective complement to the quantitative nutrient information presented in NFTs. With the notable exception of GDA-based systems, there was evidence in support of all FOPL systems, although Warning FOPLs may be particularly effective due to their ease of understanding. The findings have potentially important policy implications for several countries in the process of implementing FOPL policies, including the importance of mandated versus voluntary FOPL standards: mandated FOPLs are associated with higher levels of noticing and use, and avoid the risk of industry selectively applying FOPLs only on more nutritious pre-packaged foods.

## Supplementary Information

**Additional file 1.** Nutrition facts tables evaluated in the International Food Policy Study, by country and year.

**Additional file 2.** Noticing food labels on packages or in stores among respondents aged 10-17: 5-point Likert scale.

**Additional file 3.** Use of nutrition labels when deciding what to eat or buy among respondents aged 10-17: 5-point Likert scale.

**Additional file 4.** Self-reported understanding of nutrition label information among respondents aged 10-17: 5-point Likert scale.

## Data Availability

The datasets used and/or analysed during the current study are available from the corresponding author on reasonable request.

## Abbreviations

IFPS: International Food Policy Study
FOPL: Front-of-package nutrition label
GDA: Guideline Daily Amount
NFT: Nutrition facts table
UK: United Kingdom
US: United States
