# Supplementary material for: Awareness, use and understanding of nutrition labels among children and youth from six countries: findings from the 2019 – 2020 International Food Policy Study
Source: Int J Behav Nutr Phys Act. 2023 May 4;20:55. doi: 10.1186/s12966-023-01455-9 (PMC10157591; doi:10.1186/s12966-023-01455-9)
Supplement: Supplementary file 1 — Additional file 1. Nutrition facts tables evaluated in the International Food Policy Study, by country and year. [file 12966_2023_1455_MOESM1_ESM.docx]

**Additional File 1.** Nutrition facts tables evaluated in the International Food Policy Study, by country and year

| **Australia** | **Canada** | **Chile** |
| --- | --- | --- |
| 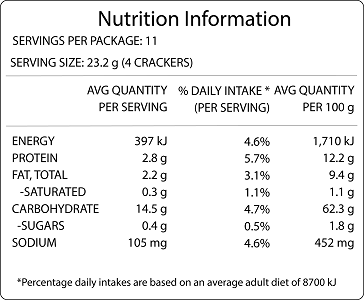 | 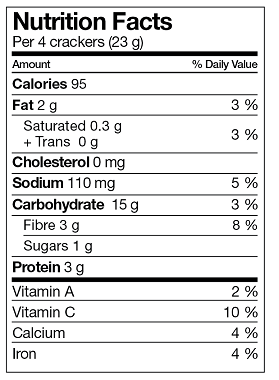 | 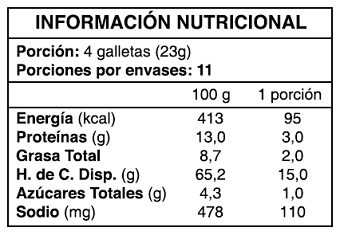 |
|  |  |  |
| **Mexico** | **UK** | **US** |
| 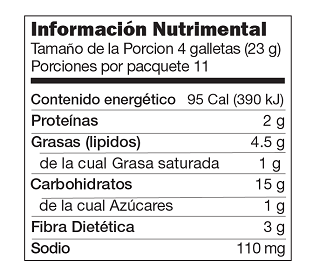 | 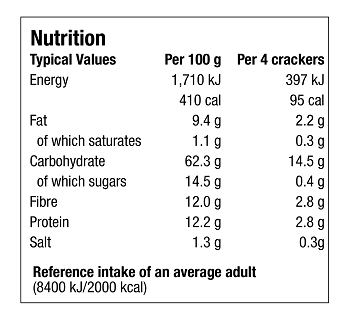 | 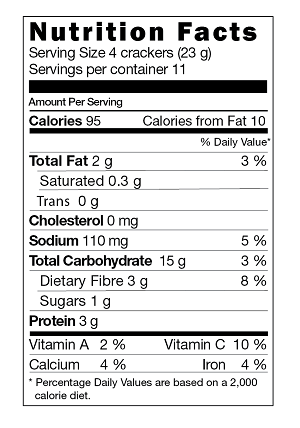 |
