# Supplementary material for: Awareness, use and understanding of nutrition labels among children and youth from six countries: findings from the 2019 – 2020 International Food Policy Study
Source: Int J Behav Nutr Phys Act. 2023 May 4;20:55. doi: 10.1186/s12966-023-01455-9 (PMC10157591; doi:10.1186/s12966-023-01455-9)
Supplement: Supplementary file 2 — Additional file 2. Noticing food labels on packages or in stores among respondents aged 10-17: 5-point Likert scale. [file 12966_2023_1455_MOESM2_ESM.docx]

**Additional File 2.** Noticing food labels on packages or in stores among respondents aged 10-17: 5-point Likert scale

|  | **Notice NFT**  Mean* (SE) | |  | **Notice FOPL**  Mean* (SE) | |
| --- | --- | --- | --- | --- | --- |
|  | **2019**  (n=10,823) | **2020**  (n=11,713) |  | **2019**  (n=10,823) | **2020**  (n=11,713) |
| **Australia** | 3.92 (.03) | 3.88 (.03) |  | 3.26 (.03) | 3.38 (.03) |
| **Canada** | 4.12 (.02) | 4.06 (.02) |  | - | - |
| **Chile** | 4.16 (.03) | 4.16 (.02) |  | 4.68 (.02) | 4.60 (.02) |
| **Mexico** | 3.88 (.03) | 4.02 (.03) |  | GDA: 3.85 (.03) | GDA: 4.04 (.03)  WL: 4.24 (.02) |
| **UK** | 3.50 (.03) | 3.48 (.03) |  | 3.63 (.03) | 3.61 (.03) |
| **US** | 4.25 (.03) | 3.97 (.03) |  | - | - |
|  |  |  |  |  |  |

NFT, Nutrition facts table; FOPL, Front-of-package label; GDA, Guideline Daily Amount; WL, Warning label

Mean score based on response on a 5-point Likert scale: Never, Rarely, Sometimes, Often, All the time
