# Supplementary material for: Awareness, use and understanding of nutrition labels among children and youth from six countries: findings from the 2019 – 2020 International Food Policy Study
Source: Int J Behav Nutr Phys Act. 2023 May 4;20:55. doi: 10.1186/s12966-023-01455-9 (PMC10157591; doi:10.1186/s12966-023-01455-9)
Supplement: Supplementary file 3 — Additional file 3. Use of nutrition labels when deciding what to eat or buy among respondents aged 10-17: 5-point Likert scale. [file 12966_2023_1455_MOESM3_ESM.docx]

**Additional File 3.** Use of nutrition labels when deciding what to eat or buy among respondents aged 10-17: 5-point Likert scale

|  | **Use NFT**  Mean (SE) | |  | **Use FOPL**  Mean (SE) | |
| --- | --- | --- | --- | --- | --- |
|  | **2019**  (n=10,823) | **2020**  (n=11,713) |  | **2019**  (n=10,823) | **2020**  (n=11,713) |
| **Australia** | 2.36 (.03) | 2.60 (.03) |  | 2.28 (.03) | 2.56 (.03) |
| **Canada** | 2.44 (.02) | 2.34 (.02) |  | - | - |
| **Chile** | 2.48 (.04) | 2.60 (.03) |  | 2.90 (.04) | 2.95 (.03) |
| **Mexico** | 2.49 (.03) | 2.52 (.04) |  | GDA: 2.42 (.03) | GDA: 2.46 (.03)  WL: 2.88 (.03) |
| **UK** | 2.29 (.03) | 2.30 (.03) |  | 2.41 (.03) | 2.34 (.03) |
| **US** | 2.52 (.03) | 2.80 (.04) |  | - | - |
|  |  |  |  |  |  |

NFT, Nutrition facts table; FOPL, Front-of-package label; GDA, Guideline Daily Amount; WL, Warning label
Mean score based on response on a 5-point Likert scale: Never, Rarely, Sometimes, Often, All the time
