# Supplementary material for: Awareness, use and understanding of nutrition labels among children and youth from six countries: findings from the 2019 – 2020 International Food Policy Study
Source: Int J Behav Nutr Phys Act. 2023 May 4;20:55. doi: 10.1186/s12966-023-01455-9 (PMC10157591; doi:10.1186/s12966-023-01455-9)
Supplement: Supplementary file 4 — Additional file 4. Self-reported understanding of nutrition label information among respondents aged 10-17: 5-point Likert scale. [file 12966_2023_1455_MOESM4_ESM.docx]

**Additional File 4.** Self-reported understanding of nutrition label information among respondents aged 10-17: 5-point Likert scale

|  | **Understand NFT**  Mean (SE) | |  | **Understand FOPL**  Mean (SE) | |
| --- | --- | --- | --- | --- | --- |
|  | **2019**  (n=10,823) | **2020**  (n=11,713) |  | **2019**  (n=10,823) | **2020**  (n=11,713) |
| **Australia** | 2.86 (.03) | 3.10 (.03) |  | 3.57 (.03) | 3.69 (.02) |
| **Canada** | 3.21 (.02) | 3.16 (.02) |  | - | - |
| **Chile** | 2.96 (.03) | 3.13 (.03) |  | 4.01 (.03) | 4.07 (.023) |
| **Mexico** | 2.99 (.03) | 3.01 (.03) |  | GDA: 3.01 (.03) | GDA: 3.01 (.03)  WL: 3.98 (.03) |
| **UK** | 2.81 (.03) | 2.86 (.03) |  | 3.26 (.03) | 3.22 (.03) |
| **US** | 3.33 (.03) | 3.46 (.03) |  | - | - |
|  |  |  |  |  |  |

NFT, Nutrition facts table; FOPL, Front-of-package label; GDA, Guideline Daily Amount; WL, Warning label

Mean score based on response on a 5-point Likert scale: Very hard to understand, hard to understand, in the middle, easy to understand, very easy to understand.
